# Case report and review of literature of a rare congenital disorder: Adams-Oliver syndrome

**DOI:** 10.1186/s12871-021-01339-0

**Published:** 2021-04-15

**Authors:** Edwin Suarez, Mia J. Bertoli, Jean Daniel Eloy, Shridevi Pandya Shah

**Affiliations:** 1 Department of Internal Medicine, White River Medical Center, Batesville, Arkansas USA; 2 Rutgers New Jersey Medical School, New Jersey Newark, USA; 3 Department of Anesthesiology, Rutgers New Jersey Medical School, Newark, New Jersey USA

**Keywords:** Difficult airway, Pediatric airway management, Seizure disorders, Adams‐oliver syndrome

## Abstract

**Background:**

Adams-Oliver syndrome is characterized by the combination of congenital scalp defects and terminal transverse limb defects. In some instances, cardiovascular malformations and orofacial malformations have been observed. Little is written with regards to the anesthetic management and airway concerns of patients with Adams-Oliver syndrome.

**Case presentation:**

A five-year-old female with Adams-Oliver syndrome presented for repeat lower extremity surgery. Airway exam was significant for dysmorphic features, such as hypertelorism, deviated jaw, and retrognathia. Video laryngoscope was utilized for intubation due to the patients retrognathic jaw, cranial deformities, and facial dysmorphism. A vein finder with ultrasound guidance was needed to place the peripheral intravenous line due to her history of difficult intravenous access. The patient was successfully intubated with slight cricoid pressure applied to direct the endotracheal tube smoothly. Surgery and recovery were both unremarkable.

**Conclusions:**

Due to varying presentations of Adams-Oliver syndrome, anesthetic and airway management considerations should be carefully assessed prior to surgery. Anesthesiologists must take into consideration possible orofacial abnormalities that may make intubation difficult. Amniotic band syndrome and other limb defects could potentially impact intravenous access as well.

## Background

According to the National Institute of Health, there are about 7000 known rare diseases. Thirty million, or one in ten, individuals in the United States are currently living with a rare disease. Most rare diseases are genetic, but some occur due to infection, allergies, or abnormalities in proliferation and degeneration. About 30 % of children suffering from rare disorders die by the age of five.

**Fig. 1:**
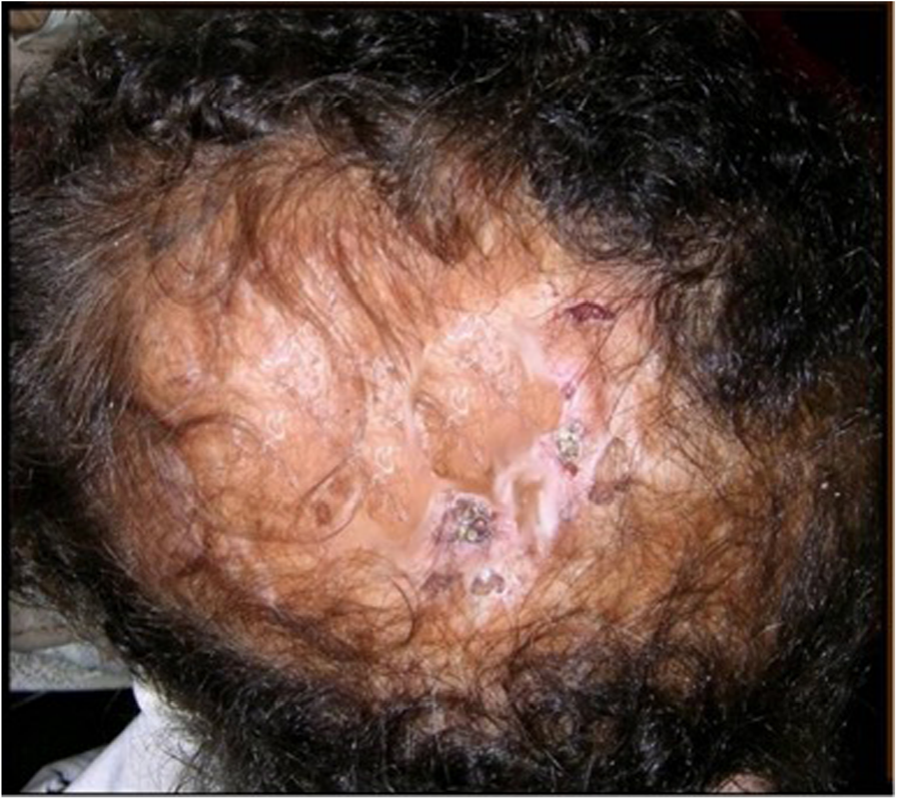
Atrophic scaring of the vertex, representing aplasia cutis congenita [2]

**Fig. 2:**
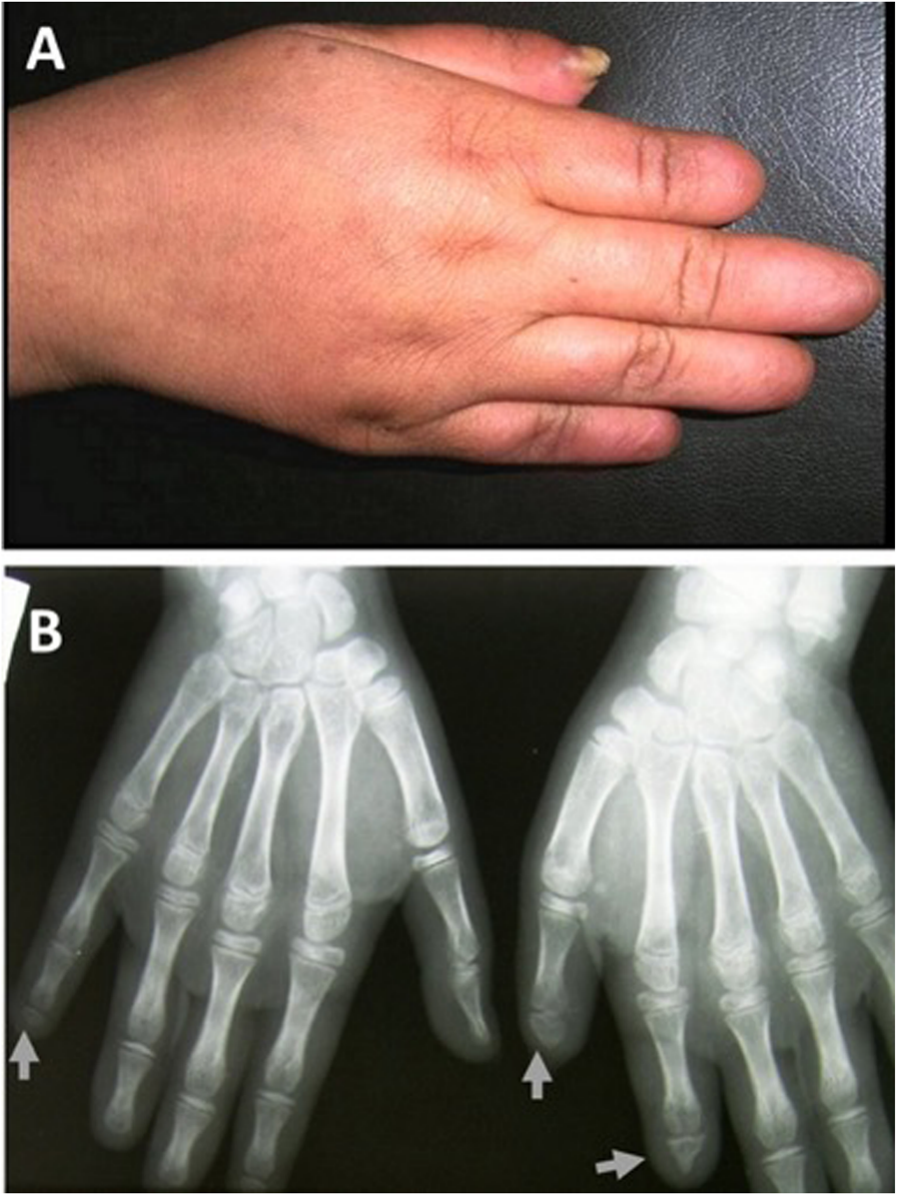
Shortening of terminal phalanges of fingers (**a**). Hand X-ray shows hypoplasia and/or complete absence of terminal phalanges (**b**) [2]

**Fig. 3:**
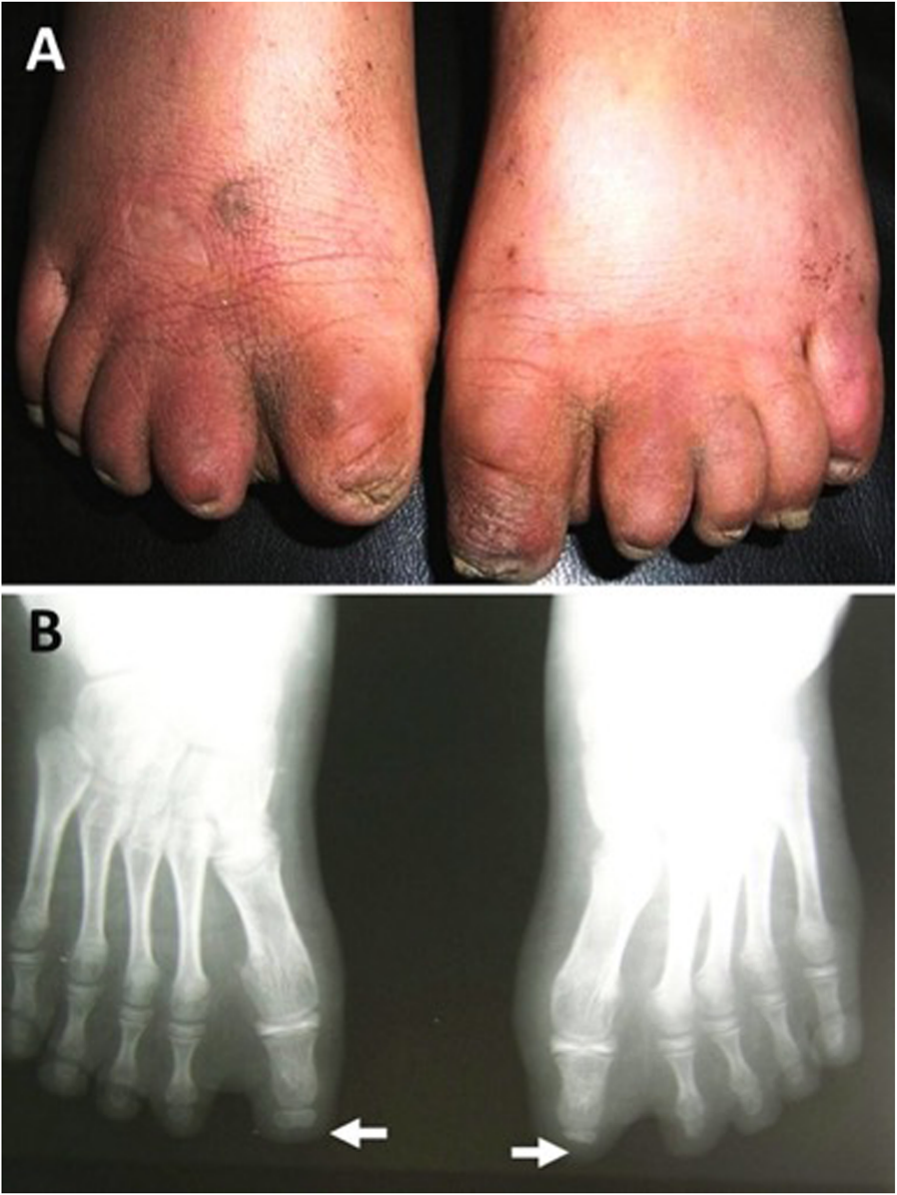
Bilateral shortening of terminal phalanges of toes (**a**) Foot X-ray shows hypoplasia and/or complete absence of terminal phalanges (**b**) [2]

Adams-Oliver syndrome (AOS) was first reported by the American pediatric cardiologist Forrest H. Adams and the clinical geneticist Clarence Paul Oliver in a family with eight affected members [1]. AOS is characterized by the combination of congenital scalp defects (aplasia cutis congenita) (Fig. 1) and terminal transverse limb defects (Figs. 2 and 3) of variable severity [2]. AOS can present with or without cutis marmorata telangiectasia congenita and it may be associated with cardiovascular or orofacial malformations [3]. Most cases are transmitted in an autosomal dominant manner, but some show autosomal recessive transmission with familial or sporadic occurrence [4]. The incidence of AOS is 0.44 cases per 100,000 live births [5]. Despite the numerous descriptions of this syndrome in the literature, little is mentioned with regards to the anesthetic management and airway concerns.

Combined with the common association of cardiac and vascular abnormalities in AOS, it is hypothesized that the spectrum of defects observed in AOS could be due to a disorder of vasculogenesis [6]. So far, the disease-causing genetic defect in AOS has not been definitively identified. We present a case illustrating the anesthetic management of a 5-year-old child with AOS with a perceived potentially challenging airway.

## Case presentation

This case involves a five-year-old Hispanic female with AOS who presented for revision of surgical release of a left clubfoot deformity. She also needed revision of right tenotomy and osteotomy for a calcaneovalgus foot deformity. She weighed 25 kg. Her history includes global developmental delay, craniosynostosis, encephalocele, hydrocephalus, amniotic band syndrome in all four extremities, syndactyly, tethered spinal cord, and uncontrolled seizures with a history of status epilepticus in infancy. Seizures were controlled at the time of presentation for surgery as last seizure was about 6 months before the presentation. Our patient had cardiac workup which was negative for congenital cardiac malformations (CCM). Her surgical history includes multiple cranial and lower extremity surgeries, and a ventriculoperitoneal shunt and revisions. Current medications include oxcarbazepine and polyethylene glycol. Airway exam was significant for dysmorphic features. This included hypertelorism, slightly deviated jaw, and retrognathia. Detailed airway exam was difficult as patient remained uncooperative and aggressive. Mother mentioned history of snoring but no tests to confirm obstructive sleep apnea were available preoperatively. Patient also had history of difficult intravenous access during previous operations.

As per mom, the child received her antiepileptic medications at home with pureed food on a routine basis. On the day of surgery, the patient did not receive her morning dose of antiepileptic medication, as her mother did not feel comfortable giving it without food. Given her aggressive behavior and history of uncontrolled seizures, premedication of 10 mg oral midazolam was given. The decision was made to have video laryngoscope and fiberoptic bronchoscope immediately available for intubation given the patient’s external airway features. Pediatric otolaryngology service was made aware of the possible need for surgical intervention and was immediately available.

Patient was calm when she arrived in the operating room. She was placed on forced air warming blanket. Standard American Society of Anesthesiologists monitors were placed. With slow inhalation induction, we were able to assist ventilation. Oral airway and some jaw thrust maneuvers were needed as anesthesia deepened. At this juncture attempts were made to establish intravenous access. Peripheral intravenous access was placed using vein finder device. After securing intravenous access, a bolus of propofol (50 mg) and 25 mcg (1 mcg/kg) dose of fentanyl was given in addition to assisted ventilation with sevoflurane to facilitate tracheal intubation. No neuromuscular blocking agent was used until the airway was secured. Direct laryngoscopy was avoided in anticipation of a difficult airway. A Cormack Lehane grade III view of the vocal cords was obtained via video laryngoscope. Endotracheal intubation was attempted with slight cricoid pressure to direct the endotracheal tube. Resistance was encountered and so the attempt was aborted. Second attempt was made with use of a bougie. Endotracheal tube then was guided into trachea over the bougie. This time the trachea was successfully intubated using 5.0 cuffed oral endotracheal tube. Placement was confirmed with auscultation and end tidal carbon dioxide. Maintenance of anesthesia was performed with sevoflurane and intermittent 10 mcg boluses of intravenous fentanyl. Rocuronium was added as surgeon requested surgical muscle relaxation. The patient remained stable during maintenance phase and was extubated without consequences. The recovery phase also remained unremarkable.

## Discussion

Planning anesthesia for a syndromic child can be challenging. As not all syndromes are well understood and discussed in literature, it is necessary to keep up with knowledge about them. There remains a major number of syndromes where mechanisms and clinical manifestations are poorly understood. AOS is one of the rare diseases which can have multisystem implications. Dr. Adams and Dr. Oliver discovered how arrested development at the embryonic level can result in a variety of malformations in humans [1]. Phenotypic variability of this syndrome results in mild to severe defects. Review of literature has shown that defects have a wide array of presentations, which include structural anomalies of the eye, palatine or auricular malformations cleft lip/palate and amniotic band syndrome resulting in deformities of extremities [1, 3, 7]. It is imperative for anesthesiologists to understand the disease and prepare in advance to plan a safe anesthetic.

Airway challenges: Aside from the surgical correction of cranial or cardiovascular defects, which inherently affect anesthetic management due to anatomical, physical, physiological, or hemodynamic concerns, little is published regarding the airway/anesthetic management of children with AOS. Specifically, those with dysmorphic features and any associated jaw or airway deformities may potentially pose a challenge for intubation. In our case, the mother was a good historian which helped us to plan our anesthetic effectively. Orofacial abnormalities that may be present in AOS patients include high-narrow palate, facial asymmetry, deep philtrum, and teeth crowding [3, 8]. Craniofacial defects have been shown to be predictors of potentially difficult intubation [9]. As such, careful assessment of the airway should be performed along with the review of a patient’s anesthetic history. Our patient had hypertelorism, slightly deviated jaw, and retrognathia. Appropriate rescue equipment should be readily available to secure the airway. Maneuvers to access the airway more easily in anatomical obstructions are head-tilt, chin-lift, and jaw-thrust [10].

Seizure activity: Epilepsy and epileptic encephalopathy have been reported as rare symptoms of AOS that are associated with the *DOCK6* mutation. Patients with this mutation not only present with variable seizure severity, but also brain malformations, ocular anomalies, and intellectual disabilities [11]. It is unclear what mutation our patient has, but she has a history of controlled seizures. Midazolam was given to our patient to manage a multitude of symptoms. We expected more cooperation during induction of anesthesia and better seizure control in perioperative period. It has been documented in literature that sevoflurane, an inhalational anesthetic routinely used in pediatric anesthesia, has stimulating effects on the brain that can potentially induce seizures [12]. Since midazolam is a short acting benzodiazepine it becomes a mainstay anesthetic inducer when there is an elevated risk of seizure. Midazolam is considered an important anesthetic as it can reliably be a component of a balanced anesthetic as well as prevent seizures in the perioperative period.

Cardiac manifestations: Congenital heart defects, seen with less frequency than limb and head defects, will further complicate the health of a newborn with AOS. Cardiac malformations seen in Adams-Oliver are diverse. No singular embryological mechanism can account for all CCMs. Our patient had a cardiac workup at birth and was not found to have any cardiac defects. About 20 % of children with AOS suffer from a congenital heart defect, so a pediatric cardiologist will need to be involved at the time of birth. Congenital heart defects, ventricular and atrial septal defects, Tetralogy of Fallot, coarctation of the aorta, and bicuspid aortic valve have been described in 15 of 112 cases of AOS [13]. The *NOTCH1, DOCK6, DLL4*, and *EGOT* mutations are associated with cardiovascular abnormalities in AOS [14].

Skeletal anomalies: Over 80 % of AOS cases involve head and limb defects, which causes these cases to be of greatest concern. While a missing finger or a missing toe is a relatively minor complication, some children with AOS will develop webbing of the hands and feet. In some cases, they suffer from a total loss of a limb or a simple shortening of the fingers and toes. In a review of a family who have 5 members affected with AOS, Kuster et al. reported that limb defects are highly variable and mostly affect the distal extremities. Congenital scalp defects and distal limb anomalies have variable inheritance patterns, but amniotic band syndrome in AOS cases is reported to be of sporadic inheritance [15]. *ARHGAP31* mutations are associated with transverse limb defects [14]. Limb defects in our patient presented with classic findings of amniotic band syndrome and syndactyly. Our patient had very small palms and very short fingers and absent distal phalanxes on the fingers.

## Conclusions

AOS is a complex disorder presenting with phenotypic variability. Possible defects include congenital scalp defects, limb defects, cardiovascular malformations, orofacial malformations, retrognathia, and many others. Our patient had a retrognathic jaw and amniotic band syndrome, with a history of difficult intravenous access. Since proper precautions were taken, the anesthetic management was without complications and the patient successfully recovered. Since there is little written about AOS and anesthesia, anesthesiologists must be aware of the possible challenges and prepare for difficult airway maintenance and intravenous access. Due to the heterogeneity in disease symptoms and multisystem implications, it is imperative for anesthesiologists to collaborate with multiple different specialties prior to anesthetic management to ensure a safe perioperative experience.

## Data Availability

N/A.

## Abbreviations

AOS: Adams-Oliver syndrome
CCM: Congenital Cardiac Malformations
